# Evaluation of a polyvalent foot-and-mouth disease virus vaccine containing A Saudi-95 against field challenge on large-scale dairy farms in Saudi Arabia with the emerging A/ASIA/G-VII viral lineage

**DOI:** 10.1016/j.vaccine.2017.10.029

**Published:** 2017-12-14

**Authors:** Nicholas A. Lyons, Anna B. Ludi, Ginette Wilsden, Pip Hamblin, Ibrahim Ahmed Qasim, Simon Gubbins, Donald P. King

**Affiliations:** a The Pirbright Institute, Ash Road, Pirbright, Woking GU24 0NF, UK; b European Commission for the Control of Foot-and-Mouth Disease (EuFMD), Food and Agriculture Organisation of the United Nations, Rome, Italy; c Directorate of Animal Resources Services, Ministry of Environment, Water and Agriculture, Saudi Arabia

**Keywords:** Foot-and-mouth disease, Vaccines, Correlates of protection, Vaccine evaluation

## Abstract

In 2015, foot-and-mouth disease (FMD) viruses of the A/ASIA/G-VII lineage emerged from the Indian sub-continent to cause outbreaks in the Middle and Near East. A factor which has been proposed to have contributed to the rapid spread of this lineage is the poor *in vitro* vaccine-match of field isolates to vaccine strains that are commonly used in the region. This study used data from outbreaks on four large-scale dairy farms using routine vaccination in Saudi Arabia, to evaluate the impact of vaccination and learn how to manage outbreaks more effectively in this setting. This evaluation also included an assessment of vaccine-induced neutralisation titres to the vaccine and field strains on a related farm with no history of FMD that employed an identical vaccination schedule. The incidence risk among exposed groups ranged from 2.6 to 20.1% and was significantly higher among youngstock (18.7%) compared to adults (7.4%). Evidence was found that local isolation of individual sick animals was more effective than whole group isolation and that subclinical infection and undetected circulation may occur on large-scale farms in Saudi Arabia, although both of these points require further evaluation. On the unaffected farm, the mean reciprocal titres for the vaccine and field strains were all above the cut-off supposed to correlate with clinical protection based on evidence from challenge studies. An estimate of vaccination effectiveness was not possible on the affected farms, but the incidence of FMD provides a more realistic estimation of the expected vaccine performance than *in vivo* studies or r_1_ value as it is based on field conditions and natural exposure. This study shows that analysis of field data from FMD outbreaks are a useful addition to more conventional challenge and *in vitro* based evaluations of vaccines and suggests further work is necessary to validate correlates of protection in field conditions.

## 1 Introduction

Foot-and-mouth disease (FMD) is a viral disease of cattle that has negative impacts for farmers in endemic countries including direct production losses and indirect losses related to implementing control measures [1]. Vaccination is one of the major tools for FMD control to mitigate the impact of clinical disease, or to reduce and eventually eliminate virus circulation as outlined in the Progressive Control Pathway for FMD control [2]. Farmers and governments dedicate large amounts of resources to purchasing and administering FMD vaccines; either for routine prophylaxis or reactively in response to an increase in exposure risk. There are a variety of documented problems with currently available FMD vaccines including antigenic mismatch between the vaccine and field strains, their relatively short shelf life, reliance on the cold chain, and a short duration of action [3], [4]. Despite these constraints, vaccines have been used successful for FMD control, especially when used alongside other zoo-sanitary measures [5].

FMD vaccines are usually evaluated either by performing challenge studies in containment facilities or by demonstrating seroconversion to antibody levels that correlate with protection in susceptible species [6]. However, these approaches have important limitations including: small sample size; use of artificial exposure methods with uncertain relevance to challenge under field conditions; only considering protection after a single dose of vaccine; challenge occurring at a fixed time point after vaccination so not accounting for waning immunity over time and typically only challenging a certain age and breed demographic. To address the issue of antigenic matching between the vaccine and field strains, heterologous challenge studies can be performed but have similar constraints to the homologous tests. There are various *in vitro* serological assays that can be used to predict cross protection. The test outlined by the OIE [6] compares post-vaccination titres to the homologous vaccine and heterologous field strain to generate a relationship coefficient (r_1_ value). Although commonly performed, this test suffers from technical variability [3] and it is unclear what level of protection is expected in field conditions for a particular r_1_ value. Other tests that have been developed including measuring IgG subtypes and antibody avidity that may improve the predicted cross-protection, though these are less frequently used and further validation is needed [7]. These combined limitations highlight the importance of performing field studies alongside *in vitro* and *in vivo* experiments to evaluate vaccines, although the results of such field studies are infrequently reported in the literature.

In 2015, viruses from serotype A (topotype ASIA, genotype G-VII, referred to as A/ASIA/G-VII), previously limited to the Indian sub-continent, emerged in Saudi Arabia, Turkey, Armenia and Iran [10]. Outbreaks due to this lineage continue to pose a risk in these countries and beyond. The results of *in vitro* vaccine matching performed at FAO World Reference Laboratory for FMD (WRLFMD) at The Pirbright Institute, UK have demonstrated a poor antigenic match to all commercially available vaccine strains, particularly those derived from the A/ASIA/Iran-05 viral lineage that are commonly used in the region [8]. However, these results need to be interpreted cautiously since previous studies have shown that high-potency serotype A vaccines may still provide clinical protection even when the vaccine-matching data is indicative of a poor match [9]. In this context, a recent heterologous challenge study with a multivalent vaccine containing A Iran-05 A Saudi-95; the latter being a vaccine strain more genetically closely related to A/ASIA/G-VII provided evidence of weak vaccine-induced protection, albeit below internationally recognised standards [8]. Furthermore, large-scale dairy farms in Saudi Arabia using regular vaccination with vaccines containing the A Saudi-95 strain have reported outbreaks of A/ASIA/G-VII [10].

The aim of this study was to evaluate the response of routine vaccination using a polyvalent vaccine containing the A Saudi-95 strain against the A/ASIA/G-VII lineage. Data will be presented from FMD outbreaks that occurred on four large-scale dairy farms located in Saudi Arabia that were regularly using such a vaccine. As part of this investigation, sera from a different farm that did not have clinical disease but which used an identical vaccination regimen were analysed to establish titres generated using routine vaccination. Variables associated with antibody levels at the individual animal level were investigated. Data from these outbreaks will be used to improve our understanding of FMD in this type of large scale production system.

## 2 Materials and methods

### 2.1 Farm backgrounds

All dairy farms were located in a central region of Saudi Arabia. All cattle were Holstein Friesian and loose housed in dry-lots according to age and lactation status with access to outside loafing areas. Lactating cows were milked four times daily and all breeding was done by artificial insemination. Both youngstock and adults were located on the same units but managed separately so considered separately. Electronic individual animal records were kept according to a unique ear tag identification including disease events. Replacement stock were either sourced from the same farm or introduced from a limited number of other farms in the same area. All bull calves were sold by around 14 days of age.

### 2.2 FMD history and vaccination

Data from FMD outbreaks on four different farms were used for this study. The outbreaks occurred between 3rd September 2015 and 3rd April 2017 and were the first reported outbreaks of FMD A/ASIA/G-VII in Saudi Arabia. Upon suspicion of an outbreak, farms notified the relevant national authorities and samples were submitted to the WRLFMD for confirmation, RNA sequencing and strain characterisation (vaccine matching). Farms instigated varying degrees of internal isolation depending on the facilities available. The date and unique identification number of animals with FMD were recorded manually and entered onto the farm management software. Animals were recorded as a case of FMD if the animal was seen salivating with any of the following clinical signs: mouth lesions, feet lesions, teat lesions, fever, reduced feed intake, and lameness. All recording was done by farm staff supervised by veterinary surgeons employed by the farms.

All farms in the study regularly used a polyvalent, killed, aqueous adjuvanted (aluminium hydroxide and saponin), NSP purified FMDV vaccine (Aftovaxpur, Merial Animal Health). The vaccine contained the following FMD virus strains: O Manisa, O-3039, A Iran-05, A Saudi-95, Asia-1 Shamir and SAT-2. A four dose primary course was given to youngstock at a target of four, five, six and seven months of age. This schedule was based on the recommendations outlined by Kitching and Salt [11], although an additional dose was included in the primary course due to reported breakdowns in young animals despite this schedule. Vaccines were given at the same time each month so that animals received their first dose between 3.5 and 4.5 months of age. Thereafter, animals were vaccinated every 105 days by being incorporated into the whole herd vaccination programme. Reactive vaccination was utilised to varying degrees either in response to FMD cases occurring on the farm or a perceived increase risk from suspected FMD in the area. Due to issues of vaccine availability and unawareness of the causal strain, this occasionally involved using a different polyvalent, oil-adjuvanted, NSP purified vaccine to that regularly used (Decivac, MSD) containing a single strain of serotype A in the A Iran-05 lineage (A-TUR-06). All vaccines were administered according to the manufacturer’s recommendations.

### 2.3 Serological sampling

In order to assess the antibody titres generated from this vaccine, a fifth farm was purposively selected that had no recent history of clinical FMD but used the same vaccine type (Aftovaxpur) and schedule. The last reported FMD outbreak on this farm was in 2008 before any of the animals currently on the farm were born. Animals may have been introduced from other farms, but were not included in the sampling. To ensure even age representation, an age-stratified sampling scheme was used. A target of 15 cattle were randomly selected for sampling from each of the following age groups: 6–12 months, 1–2 years, 2–3 years, 3–4 years, >4 years. A single group with animals of the appropriate age group was selected for sampling. Individuals were selected within each group based on the order they arrived in the handling facility. For the animals in the 6–12 month age strata, the farm had already sent samples to the WRLFMD on their own accord to monitor post-vaccination titres. Therefore, these samples were used rather that implementing re-sampling. The samples were taken between October 2014 and January 2015. All other animals were sampled in January 2016. The vaccine type and schedules that were used in cattle sampled earlier were identical so this was not considered likely to influence the results.

The farm supplied data for each animal including: individual unique identification number, date of birth, sampling date, parity, days in milk, pregnancy status, days in calf, days until next calving, days post vaccination, number of lifetime doses of vaccine received and vaccination dates.

### 2.4 Laboratory tests

All serum samples were tested for non-structural protein (NSP) and structural protein (SP) antibodies. NSP antibodies were measured using a PrioCHECK™ FMD NS Antibody kit (Prionics, Thermo Fisher) with a value greater than or equal to 50% percent inhibition being positive. All samples were run in duplicate and results designated as inconclusive if the values were either side of the positive cut-off and excluded from subsequent analysis. SP antibody titres were measured using Virus Neutralisation Tests (VNT) as described previously [12]. Two vaccine strains for serotype A (A Iran-05 and A Saudi-95) as well as a field strain from another large scale farm in Saudi Arabia of the A/ASIA/G-VII lineage (WRLFMD reference A/SAU/2/2015) were used. Virus neutralisation tests are reported as the last dilution in the series where 50% of the wells are positive [6].

### 2.5 Statistical analysis

The size of the affected groups on the study farms ranged from 3800 to 23,200 heads (Table 1). Due to these large population sizes, it could not be assumed that all animals were at equal risk of exposure to FMDV. In order to reduce biased inferences on the impact of vaccination, the disease incidence was calculated only for known exposed groups that had clinical cases within the same pen. The incidence of FMD at the management group level was based on the total number of cases in the group for the outbreak period. The denominator was the number of animals present in that management group at the start of the outbreak. Linear regression was used to estimate and compare the incidence among groups with confidence intervals based on robust standard errors that allowed for intragroup correlation.

**Table 1:** Summary of FMD outbreaks occurring on four dairy farms in Saudi Arabia confirmed as being due to FMDV lineage A/ASIA/G-VII. Adults are defined as cattle that have had at least one calf.

Study population	Total cattle	Number of groups	Date of index case	Number of groups affected (%)	Number of FMD cases (%^a^)	Incidence risk^b^ (%, 95%CI)	Time from previous vaccination to index case (days)
A (adults)	3800	24	02/09/2015	10 (41.7)	107 (2.8)	4.7 (0.0–9.7)	65
B (adults)	20,750	82	26/12/2015	12 (15.0)	144 (0.7)	2.6 (0.1–4.6)	45
B (youngstock)	14,800	218	19/09/2015	64 (29.4)	947 (6.4)	20.1 (14.3–25.9)	15
C (youngstock)	4030	50	16/10/2015	6 (12.0)	50 (1.2)	9.9 (4.2–15.7)	43
D (adults)	23,200	99	23/10/2015	34 (34.3)	882 (3.8)	9.7 (7.0–12.5)	50

a Denominator is total number of cattle within the study population.

b Incidence risk among groups that had clinical cases. 95%CI using robust standard errors accounting for intragroup correlation.

Logistic regression was used to compare the odds of being NSP antibody positive in the different age categories. Interval regression was used to analyse the VNT data, as this method allows a value to fall within defined intervals and also accounts for censoring of data when titres are below (left censoring) or above (right censoring) the thresholds of detection. To illustrate the variability, the true result is expected to lay between dilution series either side of the reported value. For example a result of 1/16 is expected to lie between 1/8 and 1/32. These boundaries are highlighted in Appendix 1. Such an approach has been shown to provide less biased estimates for dilution series data compared with other approaches, particularly when censoring is above 20% [13]. All analysis was done on the logarithm (base 10) of the reciprocal titres. All analysis was done in Stata 14.2 (StataCorp LP, Texas, USA).

## 3 Results

### 3.1 Outbreak description

Data were analysed from four farms that had clinical FMD confirmed by the WRLFMD as lineage A/ASIA/G-VII. The four farms are labelled as A, B, C and D in order of the onset date for the index case. Outbreak data are summarised in Table 1. Adults (i.e. cattle that had at least one calf) and youngstock are managed separately on all farms so were considered separately. In Farms A and D only adults were affected, whilst on Farm C only youngstock groups were affected. Farm B was the only farm to experience FMD in both adults and youngstock. Between 12% and 47% of management groups had at least one clinical case. The incidence risk among exposed groups ranged from 2.6 to 20.1%. The overall incidence risk among youngstock was significantly higher than adults (18.7% [95% confidence interval (CI): 13.6–23.9] versus 7.4% [95% CI: 5.2–9.6], respectively; P < .0001). The group level incidences did not include any further cases that may have occurred in isolation, as groups were mixed and records were not kept of individual animal movements within isolation.

**Fig. 1:**
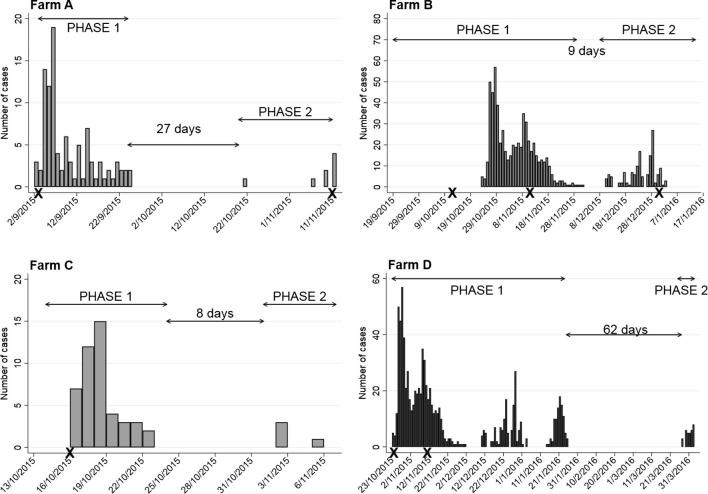
Epidemic curves for FMD cases on each of the four study farms. Also indicated are the phases of the outbreak referred to in the text and the number of days between the phases. Times of reactive vaccination are indicated by “X” on the x axis.

The epidemic curves are shown in Fig. 1. Two distinct phases of the outbreak can be seen, which is consistent with reports from the farm staff. On Farms B and C, the time period between phases were short at nine and eight days, respectively, but they were more prolonged on Farms A and D at 27 and 62 days, respectively.

Maps of affected groups on each farm are shown in Appendix 2. The spatial distribution of cases was relatively restricted on Farms A and C despite close proximity of other livestock of similar or younger ages that would be expected to be of similar susceptibility to disease. On Farm A, youngstock and adults were separated by 12 m and a hay bale barrier, yet there were no cases seen in these youngstock. On Farm C there were unaffected youngstock groups of similar ages located approximately 5 m from affected groups. In contrast on Farms B and D affected groups were relatively more widespread.

Farms isolated animals to varying degrees depending on the facilities available. All isolation units were located on the same farm site. For Farms A and C, isolation tended to be close to where the cases were identified in neighbouring pens. Farm A initially isolated individual sick adults within an isolation unit around 1 km away from the main cattle houses. After approximately ten cases were isolated, this changed to a house within the main farm area. After around 60–70 cases had occurred, animals were left within their original groups. Farm C isolated individual sick youngstock in a pen close to the affected groups. Farms B and D performed more extensive isolation. The latter moved all clinically affected adults to an isolation unit 6–8 km away. Farm B moved six groups of animals (containing clinically affected and unaffected cattle) located close to the index case to the isolation units immediately upon disease suspicion. Due to the outbreak progressing, 19 days after the index case, a row of 11 youngstock corals (containing affected and unaffected animals) were moved to the same isolation facility around 3 km away. Once in the isolation unit, diseased animals were separated from non-diseased leading to a restructuring of the groups such that epidemic curves for individual groups cannot be reliably created. Due to pressure over animal numbers, restocking of ten empty pens took place around three weeks later with cases subsequently occurring in these groups 20 days after restocking.

### 3.2 Vaccination

For each farm, the index case occurred between 15 and 65 days (mean 33.8 days) since the last herd level vaccination (Table 2). Regarding the vaccine given prior to the index case, on farms A, C and D, all animals were given the usual vaccine (Aftovaxpur, Merial). On Farm B, the adults were also given this vaccine, though some youngstock groups were given an alternative vaccine (Decivac, MSD) due to the usual vaccine not being available. The serotype A component of this vaccine differed in only including an A Iran-05 strain (A-TUR-2006) and not an A Saudi-95 strain, and having a double oil emulsion adjuvant versus the aqueous based routine vaccine containing saponin and aluminium hydroxide.

**Table 2:** Timing and type of vaccinations given to each farm relative to the onset of the index case.

Farm	Time since last vaccination at index case (days)	Vaccine used pre-outbreak	First reactive vaccination – days after index case	First reactive vaccination – vaccines used	Incidence risk^a^ (%, 95%CI)
A (adults)	65	Aftovaxpur	1	Aftovaxpur	4.7 (0.0–9.7)
B (adults)	45	Aftovaxpur	5	Aftovaxpur	2.6 (0.1–4.6)
B (youngstock)	15	Aftovaxpur and Decivac	23	Decivac	20.1 (14.3–25.9)
C (youngstock)	43	Aftovaxpur	0	Decivac	9.9 (4.2–15.7)
D (adults)	50	Aftovaxpur	1	Aftovaxpur and Decivac	9.7 (7.0–12.5)

a Incidence risk among groups that had clinical cases. 95% CI using robust standard errors accounting for intragroup correlation.

There was no apparent correlation between the incidence risk and time since vaccination prior to the index case (Table 1). On Farm B, although youngstock groups received different types of vaccine prior to the index case, this allocation was related to age with older animals receiving Decivac. Therefore calculation of the vaccine effectiveness was not possible as age could not be adjusted for in a statistical model.

All four farms implemented reactive vaccination once the index case had been observed. The timings and types of vaccinations relative to the index case are presented in Table 2 (see also Fig. 1). On Farm D, two types of vaccine were used in adults reactively but no cases were seen in the groups given Decivac so it is unclear based on clinical signs whether these groups were exposed to virus.

### 3.3 Serology

**Fig. 2:**
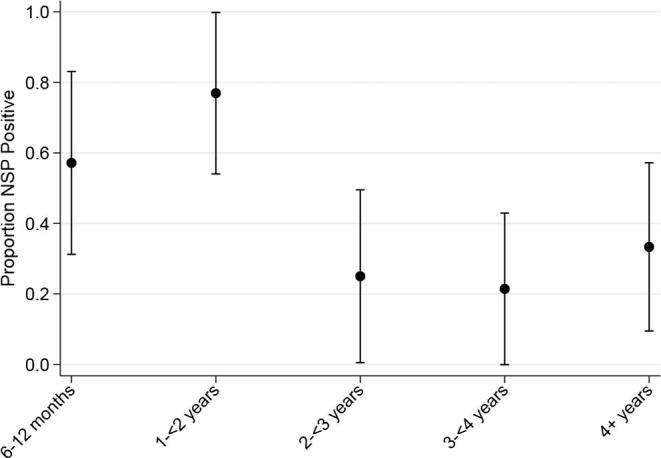
Age distribution of the proportion of NSP positive samples with 95% confidence intervals. Inconclusive results (n = 2) not included.

Data on the animals sampled for serology from an unaffected farm are presented in Table 3. Of all sera tested, 29 out of 70 (41.4%) animals were seropositive for NSP antibodies with 2 out of 70 animals being inconclusive. The age distribution of seropositivity is shown in Fig. 2. The proportion of animals with a positive result was highest in the 1–2 year age category. Logistic regression using the 1–<2 year age category as a baseline indicated that cattle in the 2–<3 years, 3–<4 years and ≥4 years age categories were at significantly lower odds of being seropositive in contrast to the 6–12 month category which was not significantly different to the baseline (Odds ratios [95% confidence intervals] 6–12 months: 0.4 [0.075–2.1]; 1–2 years: Baseline; 2–<3 years: 0.10 [0.16–0.63]; 3–<4 years: 0.082 [0.013–0.50]; ≥4 years: 0.15 [0.028–0.80]). There was no evidence that increasing number of lifetime doses was associated with an increase in NSP positivity.

**Table 3:** General information of animals sampled as part of serological study by age strata. All cattle were female and of the Holstein Friesian breed.

Variable		Age stratum (months)					
		6–12	12–24	24–36	36–48	Over 48	All animals
Number of animals sampled		15	13	13	14	15	70
Age (months; mean, range)		9.6 (8.2–11.4)	18.7 (14.6–21.6)	26.8 (24–31)	39.2 (37.4–40.8)	59.4 (51.8–65.8)	31.1 (8.2–65.8)
Parity (mean, range)		0 (0–0)	0 (0–0)	0.8 (0–1)	1.0 (1–1)	1.9 (1–2)	0.8 (0–2)
Lifetime number of doses (mean, range)		5.3 (5–6)	7.8 (7–9)	9.9 (9–11)	13.6 (13–14)	19.3 (17–21)	11.3 (5–21)
Days post vaccination (mean, range)		42.3 (29–60)	53.0 (53–53)	58.1 (53–59)	53.0 (53–53)	53.0 (53–53)	51.7 (29–60)
Days in milk at sampling	Number lactating	0	0	11	14	15	40
	Mean (range)	–	–	107.0 (22–205)	478.8 (395–533)	531.4 (356–1105)	396.3 (22–1105)
Days in calf at sampling	Number pregnant	0	11	7	13	8	39
	Mean (range)	–	160.1 (57–213)	93.7 (47–219)	211.2 (122–277)	202.1 (87–271)	173.8 (47–277)

Each sample was tested for virus neutralisation titres to the two homologous strains of serotype A in the vaccine (A Iran-05, A Saudi-95) and a field strain isolated from an affected large-scale farm in Saudi Arabia (A/SAU/2/2015). Based on interval regression, the mean log_10_ reciprocal titres for A Iran-05, A Saudi-95 and A/SAU/2/2015 were 2.84 (95% CI: 2.77, 2.92), 2.87 (95% CI: 2.82, 2.93) and 2.48 (95% CI: 2.40, 2.55), respectively. The non-overlapping confidence intervals indicate that the heterologous titres to the latter field strain were significantly lower than for the two homologous vaccine strains. However, all individual titres and their lower 95% confidence limits were above the 2.067 protection cut-off reported by Barnett et al. [14] for serotype A strains based on data from the WRLFMD in challenge experiments.

**Fig. 3:**
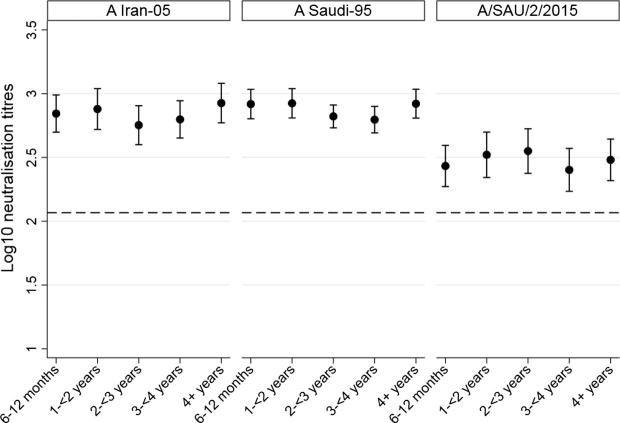
Age-specific neutralisation titres for the three FMD viruses identified at the top of the graph. Estimated by interval regression accounting for interval data and right censoring of the titres. The horizontal dashed line (titre = log_10_ 2.067) represents the cut-off thought to represent clinical protection in 95% of individuals challenged experimentally for serotype A [14].

Univariable interval regression analysis is shown in Table 4. The titres according to each age category are shown in Fig. 3. There did not appear to be any significant change in titre with age, consistent with interval regression using age at sampling as a continuous variable. Similarly, there was no evidence of an increase in titre according to the number of lifetime doses received, being NSP positive, and days in milk at calving. For A Iran-05 there was a significant decline in neutralising titres with the days post vaccination (coefficient: −0.14, 95% CI: −0.25, −0.0043; P = .005) although this effect was not seen for the other strains. Similarly, for A/SAU/2/2015, there was weak statistical evidence of a decline in neutralisation titres with the number of days in calf at sampling although the effect was small (coefficient: −0.0014, 95% CI: −0.0029, 0.00015; P = .08).

**Table 4:** Univariable interval regression analysis comparing various variables with the level of virus neutralisation titre detected. Analysis accounts for interval nature of serological data and right censoring.

Variable	FMD virus	Coefficient	95% CI	P-value
Age (months)	A Iran-05	−0.010	−0.0029, 0.0049	0.60
	A Saudi-95	−0.00024	−0.0031, 0.0026	0.87
	A/SAU/2/2015	−0.00036	−0.0046, 0.0039	0.87
Lifetime number of doses	A Iran-05	0.0056	−0.0080, 0.019	0.42
	A Saudi-95	−0.00070	−0.011, 0.0094	0.89
	A/SAU/2/2015	−0.0040	−0.015, 0.15	0.96
Time since vaccination (days)	A Iran-05	−0.014	−0.025, −0.0043	0.0050
	A Saudi-95	−0.0042	−0.011, 0.0031	0.26
	A/SAU/2/2015	−0.0016	−0.011, 0.0079	0.74
Exposure (NSP positive)	A Iran-05	0.00019	−0.0032, 0.0036	0.91
	A Saudi-95	0.00088	−0.0015, 0.0032	0.47
	A/SAU/2/2015	0.00066	−0.0031, 0.0045	0.73
Days in milk	A Iran-05	0.00021	−0.00016, 0.00058	0.28
	A Saudi-95	−1.4 × 10^−6^	−0.00022, 0.00021	0.99
	A/SAU/2/2015	−0.00024	−0.00072, 0.00025	0.34
Days in calf	A Iran-05	−0.00055	−0.0021, 0.0010	0.50
	A Saudi-95	−0.00039	−0.0014, 0.00062	0.45
	A/SAU/2/2015	−0.0014	−0.0029, 0.00015	0.077

## 4 Discussion

Detailed descriptions of disease outbreaks in endemic settings are rarely reported in the literature, but can provide useful information on the effectiveness of control measures that could be applied in endemic and FMD free settings. Challenge and *in vitro* studies are heavily relied upon for evaluating FMD vaccines. The PD_50_ or PPG measurements are based on challenge experiments with a homologous virus [6]. *In vitro* vaccine matching is based on a comparison of neutralisation titres of post vaccination sera to the field and homologous vaccine strains. An r_1_-value of 0.3 or above is used to indicate a vaccine that should provide sufficient match to confer protection [15]. There is evidence based on challenge studies using serotype A strains that if a vaccine has a sufficiently high PD_50_, a vaccine may afford protection even if the r_1_-value is less than 0.3 [9]. Vaccination schedules are typically based on serological studies using correlates of protection derived from challenge studies. Decision makers and vaccine manufacturers rely heavily upon these measures when deciding on vaccine strains, schedules and vaccination strategy. The data presented in this study should be seen as a more realistic indicator of expected protection with a vaccine of this PD_50_ and r_1_-value as they are derived from field conditions with natural exposure to FMDV.

This study presented data from four FMD outbreaks due to the viral lineage A/ASIA/G-VII that occurred on large-scale dairy farms employing frequent, regular vaccination. The vaccination schedule employed a multi-valent vaccine where the PD_50_ of each component was >6.0, and vaccination was carried out every 105 days after a four dose primary course. Although FMD outbreaks occurred, experience from incursions into this type of production system suggests the observed incidence was lower than would be expected in an immunologically naïve herd, despite the poor *in vitro* antigenic matching result [16]. Serological evidence from a non-affected farm indicates the vaccine was able to induce acceptable titres to homologous and heterologous field isolates from the A/ASIA/G-VII strain, and also that subclinical infection and virus circulation may be occurring on these farms. The lack of positive association between the number of lifetime doses received and NSP positivity suggests the latter was not the result of repeated vaccination. Evidence was also presented on isolation procedures that may be helpful for similar farms in the event of an FMDV incursion.

Empirical data relating these experimental parameters to actual FMD outbreaks in the field are lacking, with only a few examples in the literature from Turkey [17], Israel [18] and Kenya [16]. It is desirable to estimate the vaccination effectiveness when evaluating a vaccine in field conditions [19]. The vaccination effectiveness is defined as the relative reduction in the incidence of a disease outcome attributable to vaccination after adjusting for the risk of exposure. Due to the regular use of vaccination on the affected farms, there was a lack of unvaccinated animals against which to compare disease outcomes and, hence, to estimate the vaccination effectiveness, a problem highlighted previously in studies of this type of production system [16]. Similarly the reactive vaccination could not be evaluated. However, the incidence seen in exposed groups is useful for decision makers on the appropriateness of using the A Saudi-95 strain for control of outbreaks due to the A/ASIA/G-VII lineage. The occurrence of outbreaks on multiple farms implies the reason for vaccine failure was not an isolated event although it is possible that there was systematic error in vaccine delivery on these farms.

The serological study on an unaffected farm using the same vaccination protocols had the aim of providing evidence of the likely titres on the affected farms prior to the outbreak. The relatively high proportion of NSP positive cattle in the 1–<2 year age group was surprising. NSP antibodies may be present due to: FMD virus infection; maternally derived antibody prior to waning at approximately six months of age; use of non-NSP purified vaccines; or repeated use of a purified vaccine that may contain trace amounts of NSP. No clinical FMD of any serotype had been reported on this farm for several years prior to the birth of these NSP positive animals. All of the tested animals were born on the farm and were of sufficient age not to have maternally derived antibodies. It is unlikely that these results indicate antibody responses to trace amounts of NSP in the vaccine, as a linear trend in percent positivity would be expected with age (correlating correlates with the lifetime number of doses received). On this basis, the high proportion of NSP positive animals in the 1–2 year age group may indicate that subclinical or mild infection had been present in this group. The VNT levels to the serotype A strains tested in this study were not different in NSP seropositive animals indicating the exposure was likely due to another serotype. Although cattle were NSP antibody positive in this group, further groups would need to have been sampled to assess the full extent of NSP seropositivity among this age cohort on this farm. However, direct contact was possible between neighbouring groups and equipment and personnel were shared making intergroup transmission likely. The observed virus neutralising titres were all greater than a protective cut-off suggested by Barnett et al. [14], including those to the heterologous field isolate from the A/ASIA/G-VII lineage. An assessment of the neutralisation titres with age indicated that with this vaccination schedule, the titres achieved a sufficiently high level by the time the animals were 6–12 months of age, so it is unlikely that more frequent vaccination will have a positive impact on titres. Although it is possible that the titres seen on this farm were higher than in those farms that had an outbreak, this is considered unlikely because the same vaccine schedule, type, methods of cold chain maintenance and, in many cases, batch was used on these farms. Therefore, it is surprising that with these levels of antibody to the field strain that disease was seen. The protective cut-offs suggested by Barnett et al. [14] are all based on challenge studies with unclear relevance to the field regarding the route and duration of virus exposure. Serological correlates of protection for other diseases have been shown to vary with factors such as age and location [20], [21]. This is likely related to different levels of exposure seen in different settings. It is possible that protection cut-offs may vary between strains and those chosen in the study by Barnett et al. [14] may not well represent all strains present within the A serotype. Further studies would be beneficial to derive field based correlates of protection in different settings for FMD to validate the estimates based on challenge studies.

Examination of the epidemic curves on the farms revealed two distinct phases consistent with reports from the farm staff. On two farms these phases were separated by short gaps (8 and 9 days; Fig. 1) that are within the incubation period of the virus and can be explained by the virus spreading between groups on the farm. On the other two farms the gaps were longer at 27 and 62 days (Fig. 1) and may be due to prolonged circulation (under-reporting or subclinical infection) or new virus introductions. Subclinical circulation may have important implications for disease surveillance in vaccinated herds, so would benefit from further investigation.

These outbreaks also provided some insights on the potential impact of isolation on the progression on an outbreak within an affected farm. On all four farms, isolation was implemented early during the outbreak and to varying degrees depending on the resources available. On two farms, local isolation of individual animals seemed to be effective in containing the spread of virus. NSP serology may have assisted with this interpretation by providing evidence of infection in non-clinically affected groups. However, the occurrence of regular outbreaks and the presence of maternal antibody would have made interpretation difficult. Outbreaks on the other two farms were more widespread despite extensive isolation of whole groups of animals. This may indicate that the movement of whole groups of animals may present some risk of transmission within the herd and that the procedures should be modified. However, these farms were also larger in size which may have also been a factor related to the wider outbreak distribution. Further observations of field outbreaks will provide further evidence of optimal management of outbreaks to reduce the impact of incursions.

In summary, analysis of data from these outbreaks provided useful information on the potential effectiveness of different isolation procedures for limiting spread of virus and also the A Saudi-95 vaccine for protecting against lineage A/ASIA/G-VII strains. There is also evidence that the usual serological correlate of protection may need further investigation to assess its relevance in different field conditions.
