# Supplementary material for: Evaluation of a polyvalent foot-and-mouth disease virus vaccine containing A Saudi-95 against field challenge on large-scale dairy farms in Saudi Arabia with the emerging A/ASIA/G-VII viral lineage
Source: Vaccine. 2017 Dec 14;35(49Part B):6850–7. doi: 10.1016/j.vaccine.2017.10.029 (PMC5723706; doi:10.1016/j.vaccine.2017.10.029)
Supplement: Supplementary data 1 [file mmc1.docx]

***Appendix 1***

Assumed upper and lower bounds for reported virus neutralisation titres. Mid-point titres between bounds are calculated using logarithms base 10.

| Dilution | Titre | Lower bound | Upper bound |
| --- | --- | --- | --- |
| 1/22 | 1.34 | 1.04 | 1.65 |
| 1/32 | 1.51 | 1.20 | 1.81 |
| 1/45 | 1.65 | 1.34 | 1.95 |
| 1/64 | 1.81 | 1.51 | 2.11 |
| 1/90 | 1.95 | 1.65 | 2.25 |
| 1/128 | 2.11 | 1.81 | 2.41 |
| 1/178 | 2.25 | 1.95 | 2.55 |
| 1/256 | 2.41 | 2.11 | 2.71 |
| 1/355 | 2.55 | 2.25 | 2.85 |
| 1/512 | 2.71 | 2.41 | 3.01 |
| 1/708 | 2.85 | 2.55 | 3.15 |
| 1/1024 | 3.01 | 2.71 | ∞ |
| ≥1/1413 | 3.15 | 2.85 | ∞ |
