# Supplementary material for: Evaluation of a polyvalent foot-and-mouth disease virus vaccine containing A Saudi-95 against field challenge on large-scale dairy farms in Saudi Arabia with the emerging A/ASIA/G-VII viral lineage
Source: Vaccine. 2017 Dec 14;35(49Part B):6850–7. doi: 10.1016/j.vaccine.2017.10.029 (PMC5723706; doi:10.1016/j.vaccine.2017.10.029)

***Appendix 2***

Schematics of farms infected with FMDV showing the spatial distribution of affected groups. Unless otherwise stated, red indicates houses with FMD cases and dashed lines indicate the location of milking parlours.

**Farm A**

Grey colour indicates position of youngstock groups.


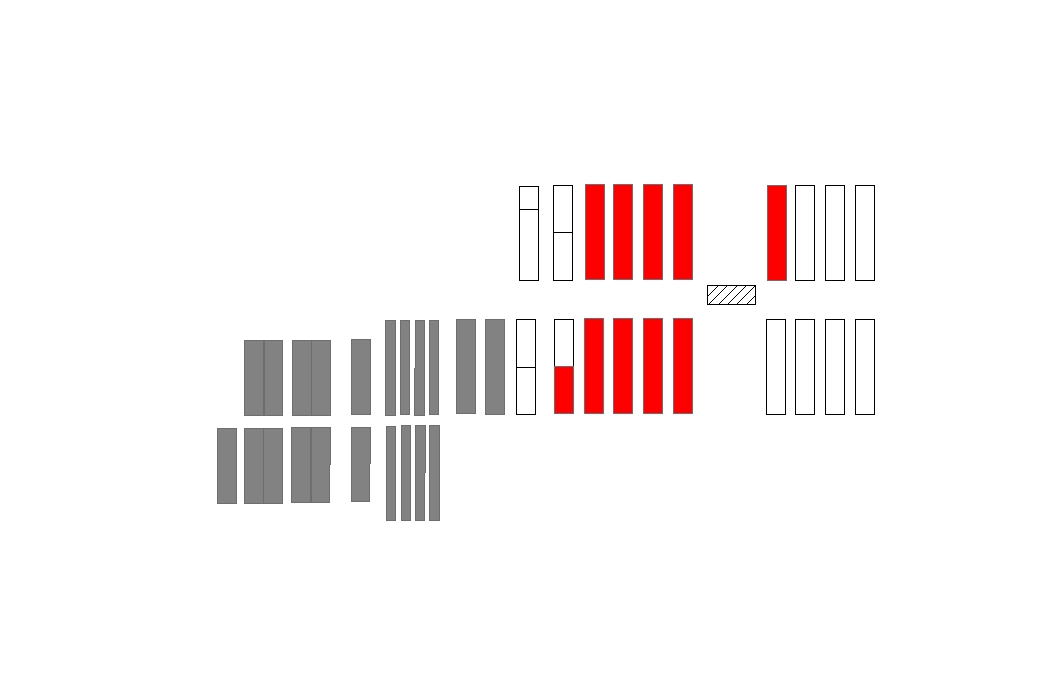


**Farm B (adults)**

**
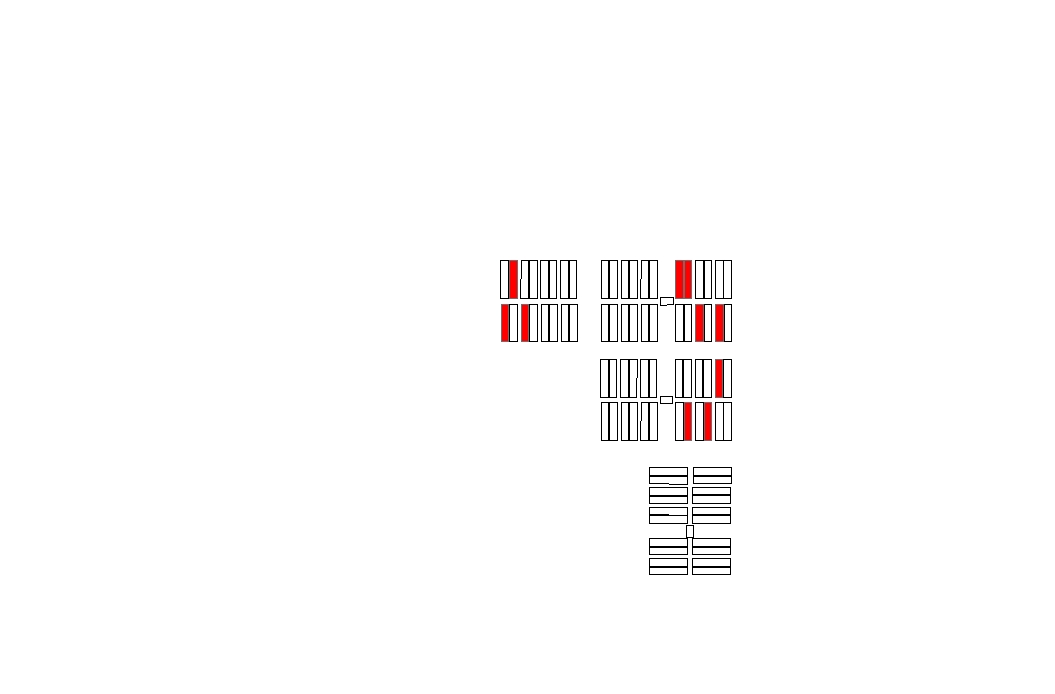
**

**Farm B (youngstock)**

**
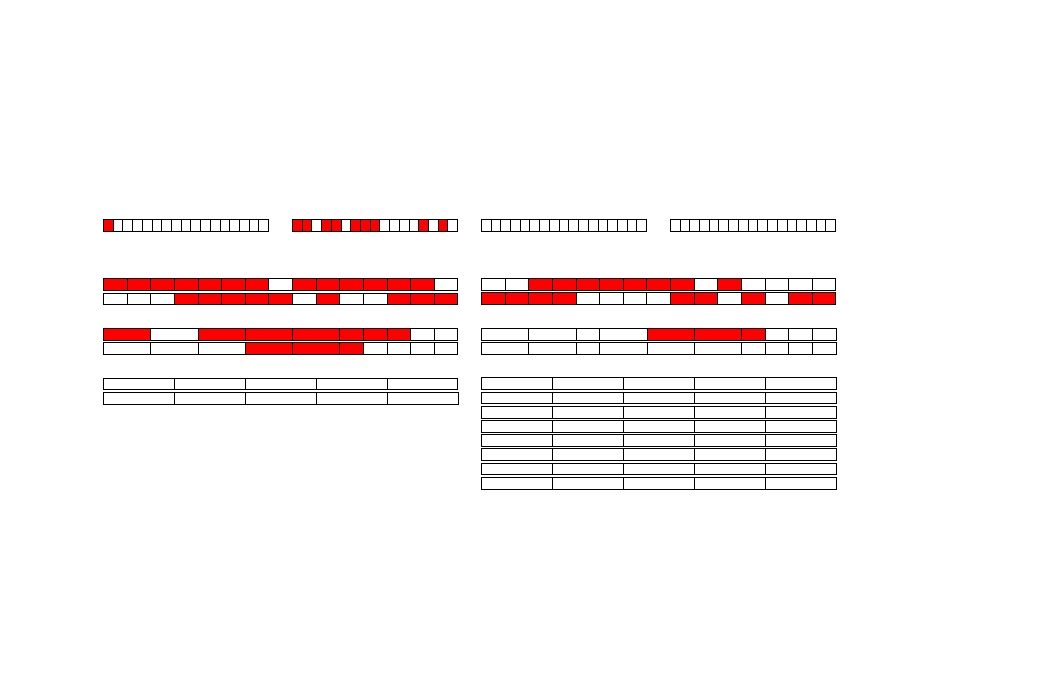
**

**Farm C**

Black colour indicates empty corrals. Dashed line shows isolation pen.

**Farm D**


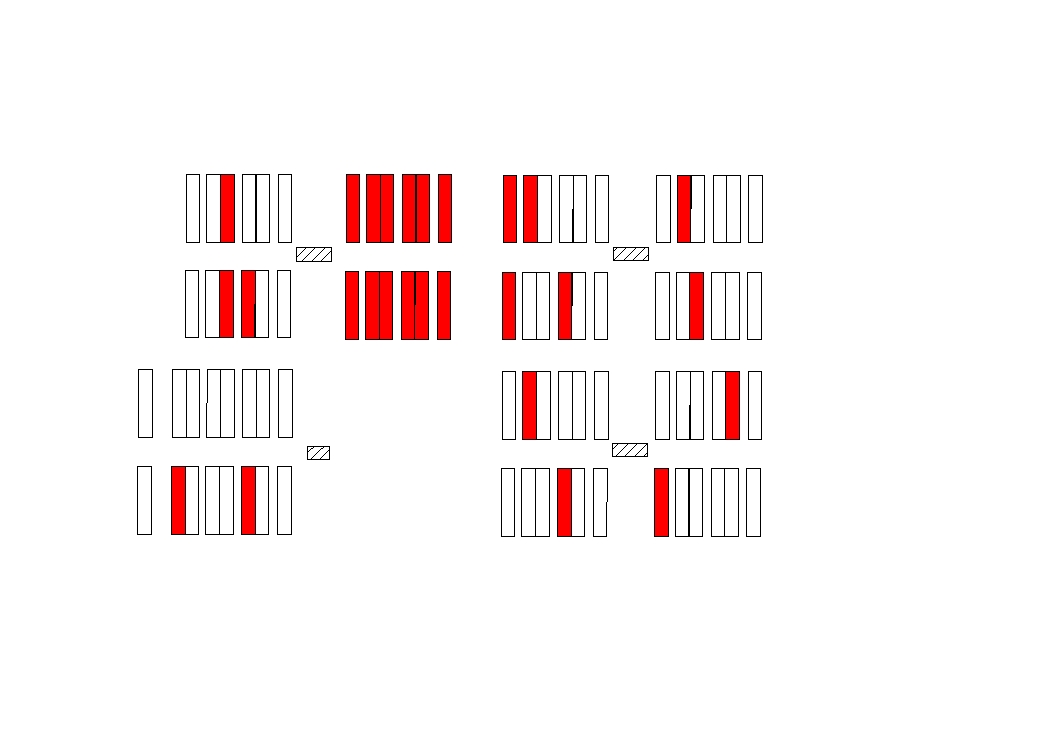

Supplement: Supplementary data 2 [file mmc2.docx]
